# Rfam 12.0: updates to the RNA families database

**DOI:** 10.1093/nar/gku1063

**Published:** 2014-11-11

**Authors:** Eric P. Nawrocki, Sarah W. Burge, Alex Bateman, Jennifer Daub, Ruth Y. Eberhardt, Sean R. Eddy, Evan W. Floden, Paul P. Gardner, Thomas A. Jones, John Tate, Robert D. Finn

**Affiliations:** 1 HHMI Janelia Farm Research Campus, Ashburn, VA, USA; 2 European Molecular Biology Laboratory, European Bioinformatics Institute (EMBL-EBI), Wellcome Trust Genome Campus, Hinxton, Cambridge, UK; 3 Biomolecular Interaction Centre, School of Biological Sciences, University of Canterbury, Christchurch, New Zealand

## Abstract

The Rfam database (available at http://rfam.xfam.org) is a collection of non-coding RNA families represented by manually curated sequence alignments, consensus secondary structures and annotation gathered from corresponding Wikipedia, taxonomy and ontology resources. In this article, we detail updates and improvements to the Rfam data and website for the Rfam 12.0 release. We describe the upgrade of our search pipeline to use Infernal 1.1 and demonstrate its improved homology detection ability by comparison with the previous version. The new pipeline is easier for users to apply to their own data sets, and we illustrate its ability to annotate RNAs in genomic and metagenomic data sets of various sizes. Rfam has been expanded to include 260 new families, including the well-studied large subunit ribosomal RNA family, and for the first time includes information on short sequence- and structure-based RNA motifs present within families.

## INTRODUCTION

Rfam is a database of non-coding RNA (ncRNA) families. Each family is composed of a multiple sequence alignment (MSA) of a representative set of sequences that includes consensus secondary structure annotation (where appropriate), a covariance model (CM) of the sequence and structure conservation of the family built from the MSA, and a set of putative homologues identified in a sequence database derived from the European Nucleotide Archive (ENA) (1). The Infernal software package (2) is used to build CMs from the alignments (termed seed alignments) and to search those CMs against the ENA-derived database (termed Rfamseq) to identify homologues. An Infernal search returns a list of high-scoring hits in the database and corresponding bit scores, which indicate how well each hit matches the family CM. Rfam curators define a bit score threshold for each family (the gathering threshold), which separates the lowest scoring presumed true homologue from the highest scoring presumed non-homologue, using expert knowledge of the family and taxonomic information of the matched sequences. In addition to ncRNA annotation, Rfam assigns a type for each family (such as Gene, *cis*-regulatory element, etc.), provides cross-references to the literature supporting each family, as well as to other relevant resources, such as the Protein Data Bank (PDB) (3), mirBase (4) and the ENA. We also add Gene Ontology and Sequence Ontology terms for each family (5) and text-based descriptions of each family are provided via Wikipedia entries (6,7).

Rfam is a large and diverse source of ncRNA annotation that includes information on many types of ncRNAs across all three domains of life and viruses. For RNA biologists seeking information on one or more RNA families, Rfam provides sequences, alignments, CMs, trees and secondary structure images. The set of Rfam CMs and score thresholds may also be downloaded and used with Infernal to identify new family members in other sequence databases and for annotating ncRNAs in genomes or metagenomes. The current release, Rfam 12.0, was made public in September 2014 and contains 2450 entries. In this article we discuss new features and changes we have made to the Rfam production pipeline and how they affect users.

## UPGRADE TO INFERNAL 1.1

For all previous releases (0.1–11.0), Rfam has annotated RNAs in Rfamseq using a two-step, Basic Local Alignment Search Tool (BLAST)—and Infernal-based homology search pipeline. In the first step, sequences from the seed alignment were used as BLAST queries against Rfamseq, and subsequences including significant hits plus some surrounding nucleotides were written to new sequence files called ‘mini-databases’. In the second step, the mini-databases were re-searched with a CM using the *cmsearch* program of Infernal. This two-step process was employed because CM methods are computationally expensive but significantly more powerful at RNA homology detection than BLAST (8), due to the fact that CMs score both conserved sequence and secondary structure while BLAST scores only sequence conservation. Ideally we would use *cmsearch* without BLAST-filtering because of its superior homology detection ability, but prior to Rfam 12.0 *cmsearch* was simply too slow to be practical. In 2013, version 1.1 of Infernal was released which accelerates CM searches about 100-fold relative to version 1.0, by using a profile hidden Markov model (HMM) filtering scheme based on the HMMER3 software package. This makes it fast enough to obviate the need for BLAST filters (2,9–10). Infernal 1.1 also offers improved handling of truncated RNA sequences (11) which are common in data sets of sequencing reads, such as those created by metagenomics experiments.

We have rewritten the Rfam family building and production pipeline to use Infernal 1.1. The new system offers two important advantages over the previous one. First, the input- and output-intensive mini-database creation step is no longer necessary. Secondly, because profile HMMs have been shown to be more sensitive for RNA homology search detection than BLAST (8), more true RNAs should survive the filtering step to be subsequently evaluated by the CM. This second advantage should improve search sensitivity and lead to an increase of the number of annotated homologues for many families. Changing to the new filtering strategy significantly affects the search results for nearly all families. Consequently, it necessitated the laborious task of re-thresholding each family in the database for Rfam 12.0 (see below).

To compare the new and old filtering strategies in terms of speed and ability to identify homologues, we searched 200 randomly chosen families from Rfam 12.0 against the 270 Gb Rfamseq database using both strategies. To compare the two strategies as directly as possible, we changed only the search methodology (BLAST plus Infernal 1.0 versus Infernal 1.1), and used identical seed alignments, CM parameters and score thresholds for both strategies, all of which derived from Rfam 12.0. Table 1 includes a summary of the results, including summed statistics over all 200 families as well as per-family statistics for 15 families, chosen as the top five, middle five and bottom five families as ranked by number of hits found by the new strategy. In terms of speed, the two approaches are very similar on average: the new Infernal 1.1-based system required 4222 total CPU hours while the old BLAST- and Infernal 1.0-based system required 4070 total hours, but vary greatly on a per-family basis, with a range from about 70-fold faster for the old strategy (RF01589, not in Table 1) to about 70-fold faster for the new strategy (RF00162). This variation is mostly due to a key difference between the two filtering strategies: the old strategy used multiple seed sequences as BLAST queries, so run times will be increased for families with larger numbers of seed sequences, whereas the new strategy uses a single query profile HMM for all families.

**Table 1.** Comparison of the old Rfam 11.0 BLAST and Infernal 1.0 search strategy versus the new Rfam 12.0 Infernal 1.1 search strategy for 15 of 200 randomly chosen families

Accession	Family ID	Length (nt)	#of seed seqs	Time new (h)	Time old (h)	Time (old/new)	New total hits	Old total hits	New unique hits	Old unique hits
Top five families										
RF00028	Intron_gpI	251	12	125.0	357.2	2.8	71 433	60 264	11 175	1
RF00026	U6	104	188	31.2	181.1	5.8	66 517	62 174	4367	14
RF00003	U1	166	100	11.6	64.0	5.5	15 770	14 867	904	1
RF00162	SAM	108	433	8.3	590.0	70.8	4905	4797	108	0
RF00050	FMN	140	144	17.1	169.9	23.9	4381	4306	76	1
Middle five families										
RF01426	snoR126	101	4	40.3	7.3	0.2	78	66	12	0
RF01252	snR5	196	11	41.1	9.8	0.2	76	72	4	0
RF00544	snopsi28S-3327	143	14	11.3	15.1	1.3	75	74	1	0
RF00439	SNORD87	85	10	26.8	12.6	0.5	75	74	1	0
RF01537	TB11Cs2H1	70	7	5.8	7.3	1.3	74	73	1	0
Bottom five families										
RF01439	S_pombe_snR36	164	2	25.0	1.7	0.1	5	2	3	0
RF01448	S_pombe_snR93	143	2	11.0	1.5	0.1	4	3	1	0
RF00967	mir-281	83	2	6.0	2.6	0.4	4	4	0	0
RF00925	MIR1027	142	2	20.4	1.6	0.1	3	3	0	0
RF01576	DdR8	88	2	10.4	1.6	0.2	2	2	0	0
all 200	-	-	-	4222.2	4069.8	0.96	201 814	179 681	22 312	53

The top five, middle five and lowest five families are shown, as ranked by number of hits found above Rfam GA thresholds using the new search strategy. Identical Rfam 12.0 score thresholds and CM parameters were used for both the new and old strategies (new: Rfam 12.0 CM file in Infernal 1.1 format; old: Rfam 12.0 CM file converted to Infernal 1.0 format using Infernal 1.1's *cmconvert* program). For each family, columns 1–4 include the Rfam accession, family identifier, model length in nucleotides and number of sequences in the seed alignment, columns 5–7 report on the running time for the new strategy in hours, old strategy in hours and the ratio of the running time (old/new), respectively, columns 8 and 9 report the number of hits found above the per-family Rfam 12.0 thresholds for the new strategy and old strategy, respectively; column 10 reports the number of unique hits found by the new strategy and not the old, and column 11 reports the number of unique hits found by the old strategy but not the new. A unique hit is defined as a hit found by one strategy for which none of the hits found by the other strategy overlap by ≥1 nucleotides on the same strand. The 200 families were randomly chosen from the set of 2190 families that exist in both Rfam 12.0 and Rfam 11.0, the last release for which the old strategy was used. Initially, MIR1122 (RF00906) was included in the 200, but we replaced it with another random choice (SNORD97, RF01291) after learning that MIR1122 is clearly related to a MITE (miniature inverted-repeat transposable element) in plants and that the curators at the microRNA database mirBase (4) suspect it may not be a true miRNA gene. If the family is removed from mirBase, it will also be removed from Rfam.

Our test results demonstrate that the new search strategy is able to find many more RNA homologues than the old one. In total, the new strategy identified 201 814 total RNAs, 22 312 of which were unique hits not found by the old strategy. The old strategy found 179 681 total hits, only 53 of which were not found by the new strategy. Of the 22 312 RNAs found only by the new strategy, 7319 (33%) were truncated hits (non-full-length hits that terminate at one or both ends of a database sequence) indicating the improved ability of Infernal 1.1 to handle partial sequences will have a significant impact on Rfam annotations. For 74 of the 200 families, the new strategy identified at least 1 unique hit that was not found by the old strategy, while the old strategy found unique hits not found by the new strategy for only 7 families. For 126 families, both the new and old strategies found the exact same set of hits (note that while not all corresponding hits had identical sequence boundaries, each hit found by one strategy overlapped by at least 1 nucleotide on the same strand with a hit from the other strategy).

We believe the vast majority of the novel hits found by the new strategy represent true homologues as opposed to false positives. As described more below, while curating Rfam 12.0, the new strategy search results for each family were manually examined to determine the gathering score threshold. All hits scoring above this threshold are considered true by our curators. It is these Rfam 12.0 thresholds, as well as Rfam 12.0 CMs, that were used in our tests for both the old and new search strategies.

Rfam physically moved from the Wellcome Trust Sanger Institute to the European Bioinformatics Institute (EBI) in 2013. Along with the update to Infernal 1.1, the move to EBI motivated us to overhaul and streamline the entire Rfam production pipeline codebase. In addition to improving RNA homology detection sensitivity, the new production pipeline has been parallelized, such that most computation is performed on a per-family basis. As much data as possible (such as the secondary structure images, tree generation and sunburst species distributions) are generated at the point of family creation or modification, which can occur at any point during the release cycle. The previous production pipeline generated the same data at the time of making the release, using a more linear process that required a large amount of compute. Because we now spread this computation out during the family curation period between releases, releases should become simpler. Another important change in Rfam 12.0 is that we no longer provide alignments of all Rfamseq hits for each family (previously termed full alignments), because these alignments continue to grow in size and many are so large that they are impractical to distribute (e.g. the tRNA alignment of 1.7 million sequences is more than 3 Gb).

## RFAM 12.0: NEW FAMILIES AND CHANGES TO OUR DATA

Rfam 12.0 was released in September 2014. It contains 2450 families and annotates 19 623 515 sequence regions from the standard and whole genome shotgun data classes of ENA release v110. Since the previous release (11.0), 18 families have been removed. Most of these modelled intronic regions of long ncRNAs (lncRNAs), and two others were for families so short (21 and 22 nucleotides) that we were unable to build a CM capable of detecting homologues with statistical significance in Rfamseq. We have added 260 new families in Rfam 12.0. The majority of the new families are bacterial small RNAs, such as the AfaR small RNA (RF02515) found in *Escherichia coli* (12) and the MtlS small RNA (RF02268) found in the *Vibrionaceae* family of Proteobacteria (13). The new Infernal 1.1-based search pipeline makes it practical for the first time to include the well-studied large subunit ribosomal RNA (LSU rRNA), which had been a long-standing and glaring omission from the database because the old BLAST- and Infernal 1.0-based search strategy required an unreasonable amount of CPU time for this long RNA (∼3000 nt) which has hundreds of thousands of homologues in Rfamseq. We have added archaeal, bacterial and eukaryotic LSU models (RF02540, RF02541, RF02543) in Rfam 12.0. These are the three longest RNAs in the database and they are among the largest Rfam families, with the eukaryotic, bacterial and archaeal LSU families having 268 892, 204 053 and 62 367 members, respectively.

### Adjustments to our curator-defined gathering thresholds for use with Infernal 1.1

The upgrade to Infernal 1.1 had a large impact on search results for almost all families, making it necessary to re-examine the score thresholds for all families. This manual process required a considerable amount of curator time spent redefining the gathering threshold that determines the set of sequences that are considered true homologues. Of the 2190 families present in both Rfam 11.0 and 12.0 releases, the absolute number of family members increased for 804 families as a result of the combination of re-thresholding and the new Infernal 1.1-based search strategy, decreased for 836 families, and 550 families had no change in the number of sequences they contained.

These numbers seem to contradict the earlier comparison of the old and new search strategies summarized in Table 1, which suggests that many more families should have gained rather than lost family members due to increased sensitivity from Infernal 1.1. This apparent discrepancy can be explained mainly by increases in family gathering thresholds between Rfam 11.0 and 12.0 for 1654 of the 2190 families, which causes a family to lose any hits that scored in the range between the old and new threshold. Of the 836 families that had fewer family members in Rfam 12.0 than in 11.0, the gathering threshold was raised for 830 of them. Thresholds were increased to cope with higher numbers of non-homologous sequences surviving the Infernal HMM filters compared with the old BLAST filters.

A notable exception to the general increase in gathering thresholds is the small subunit ribosomal RNA (SSU rRNA) families (RF00177, RF01959 and RF01960). We significantly *lowered* the thresholds for these families to improve coverage on partial SSU rRNA sequences which are common in Rfamseq, and consequently the number of sequences in these families increased greatly (e.g RF00177 had 7429 sequences in Rfam 11.0 and has 3 707 732 in Rfam 12.0). The improved ability of Infernal 1.1 to detect truncated sequences along with manual inspection has made us confident that these new sequences are indeed SSU rRNA and not false positives.

### Sequence-only consensus models for lncRNAs

LncRNAs are arbitrarily defined as non-protein coding transcripts longer than 200 bp (14). Since Rfam 11.0, we have included families which model conserved regions of lncRNAs, and release 12.0 includes 216 families of this type. We have removed secondary structure annotation from all of our lncRNA families because there is as yet little evidence to support secondary structure conservation in lncRNAs. Because the CMs for these families include zero base pairs, they are essentially equivalent to sequence-only based profile HMMs (15).

### Clans

Rfam has a quality assurance measure that prevents any one nucleotide in Rfamseq from being annotated by more than one family. This can be problematic for some large, diverse families like SSU rRNA or the signal recognition particle (SRP) RNA, for which it has been necessary to construct multiple Rfam models to effectively identify all homologues. Homologues of such families are often scored above threshold by more than one of these models, violating the single-family annotation quality assurance measure. To handle these cases, the concept of clans was introduced in 2010 (release 10.0) (7), whereby homologous families, such as the four different SSU rRNA families, are grouped together into a clan and Rfamseq sequence regions are allowed to be annotated by more than one family in a clan. To reduce the amount of annotation duplication by families within the same clan, we now undertake a process called ‘clan competing’. This process identifies two matches belonging to a single clan where there is greater than 50% overlap of the length of the shortest match and retains only the match with the lowest *E*-value, discarding the other match from the corresponding family match list.

## MOTIFS IN RFAM

### RNA motifs

Recently, a collection of RNA motifs was established by Gardner and Eldai (unpublished), termed RMfam. In this case, a RNA motif is an RNA sequence and/or secondary structure that can be found in a number of different RNA families. The RMfam collection, currently 34 models, represents commonly found structural motifs (e.g. GNRA tetraloop), short functional sequence motifs (e.g. Shine–Dalgarno sequences) and RNA–protein interaction motifs (e.g. the CsrA/RsmA binding motif). These models have been sourced from a combination of published literature, online databases and *de novo* discovery. While motifs are difficult to reliably identify in large sequence data sets simply due to their small size, it is less challenging to detect them in a smaller data set of known RNA sequences, such as Rfam. Together with RMfam, we have systematically identified instances of RMfam entries within Rfam families. In line with Rfam families, each motif is assigned a unique accession number (e.g. RM00001) and supporting information pertaining to the motif, such as PDB, structures and literature references is collected. Rather than have an independent RMfam website, the RMfam data has been subsumed by Rfam, and released for the first time in version 12.0.

### Annotating Rfam families with motifs

**Figure 1.**
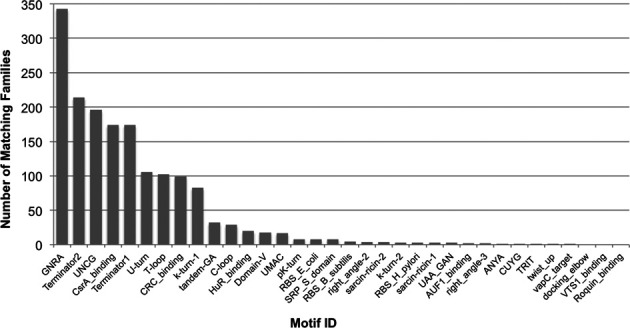
Number of Rfam family matches for each of the 34 RMfam motifs.

Using the motifs described above, the seed sequences of each Rfam family were annotated, providing an overview of the motifs in a given family. Owing to the relatively small size of the motif models, it can be difficult to distinguish the true instances of motifs from spurious matches. To alleviate this, a series of heuristic rules for annotating families with RNA motifs was implemented. For each Rfam family, the seed sequences were made non-redundant (by filtering sequences more than 90% identical). These filtered seed sequences were scanned with *cmscan* (Infernal v1.1) against the motif CM library, using the gathering threshold set for each motif. The set of matching motifs were filtered to minimize false-positive annotations by removing motifs for which less than 2 or less than 10% of seed sequences scored better than the gathering threshold. These parameter settings have been tested on a curated list of RNA families that are reported in the literature to contain motifs, including CsrB (RF00018) and RsmY (RF00195) each of which hosts multiple CsrA binding sites (16,17) as a positive control. Shuffled versions of each Rfam seed alignment (using shuffle-aln.pl, from the RNAz package (18)) were used as a negative control. This benchmark established that the sensitivity and specificity for each family is reasonable (0.93 and 0.76, respectively). A manuscript describing these results in detail is in preparation. The surviving set of motifs were re-scanned against all sequences in the complete (non-filtered) family seed alignment. Seed alignments were marked up with matching motif annotations and summary statistics for each family–motif pair were generated. Statistics include the fraction of seed sequences in a family which match a motif and the sum of bit-scores for the matches. A total of 758 families or 30% of the families in Rfam 12.0 had at least one matching motif model. Figure 1 shows the number of Rfam families annotated by each RMfam.

Several of the RMfam motifs are not local in that not all functional positions of the motif are contiguous with respect to the sequence. The internal loop motifs, such as the kink-turns (RM00010 and RM00011) (19), the Sarcin-Ricin (RM00018 and RM00019) (20) and the tandem G•A (RM00021) (21) are examples of non-local motifs, which pose a challenge to CM-based annotation because the nucleotide distance between the two halves of an internal loop is not guaranteed to be small. However, in practice, the distance between internal RNA contacts is small, even for the very long ribosomal RNA families (22) and as a result the CMs are able to annotate many of the known internal loop motifs. For the asymmetric internal loops, at least two CMs are required in order to encapsulate the two possible directions of each motif (e.g. k-turn-1 and k-turn-2, RM00010 and RM00011).

### Visualizing motifs in families

**Figure 2.**
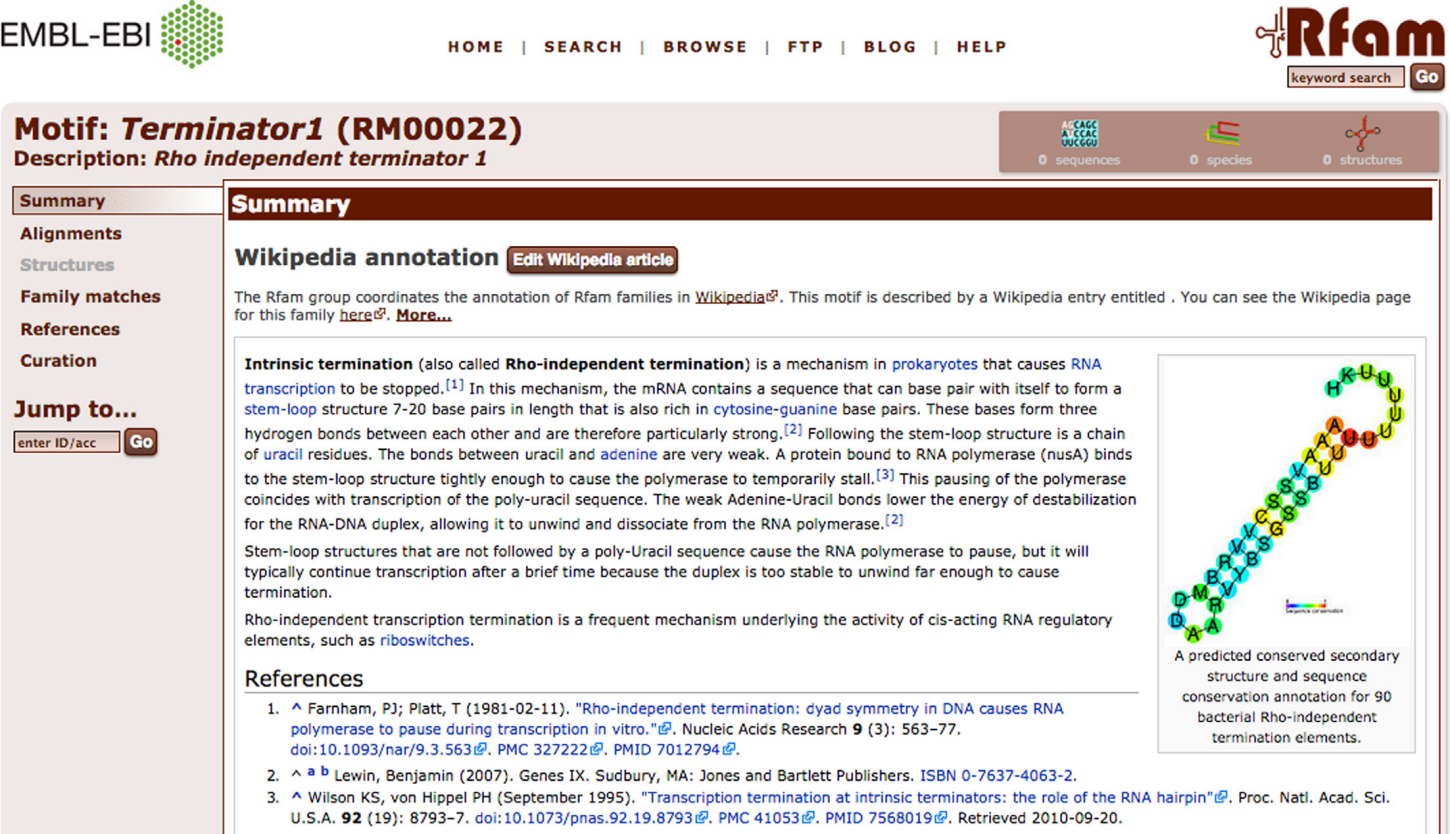
Overview of the motif page for RM00022, the Terminator1 motif, on the Rfam 12.0 website. As in family and clan pages, tabs on the left-hand side allow the user to access different information for each motif.

**Figure 3.**
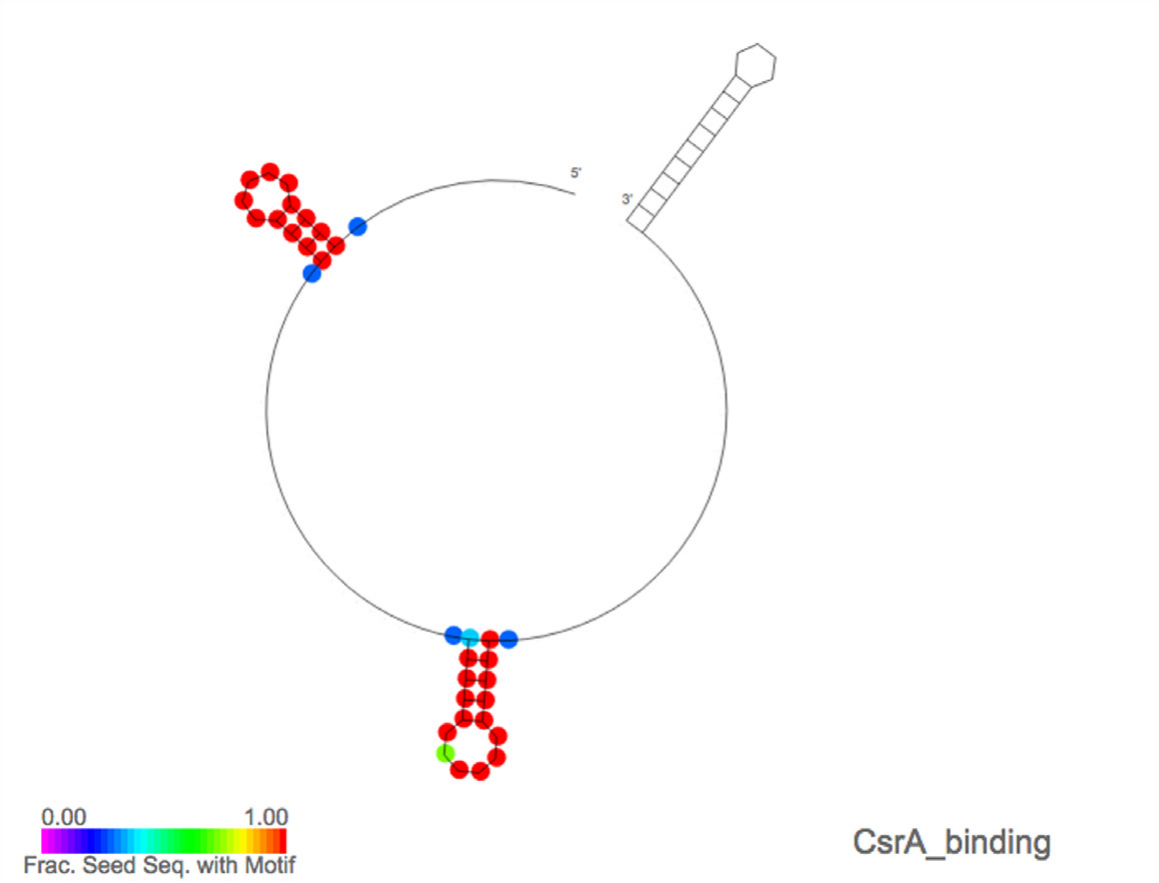
Screenshot of the secondary structure representation for the RsmY RNA family (RF00195) with the annotation for the CsrA binding motif (RM00005) overlaid. Positions in red indicate that all the seed sequences at that position are found to contain the motif while other colours represent fewer sequences having matches at that position. The CsrA protein is a homo-dimeric, RNA binding protein. Each CsrA binds a specific RNA motif that is characterized by a short hairpin that hosts a GGA subsequence, these motifs generally occur in pairs. The CsrA-binding sRNAs, like RsmY, generally sequester excess copies of CsrA which would otherwise bind mRNAs and inhibit translation (23). Therefore, the expression of these sRNAs is a rapid way of altering expression levels for a potentially large network of proteins (24).

On the Rfam website, information for each motif can be accessed via individual motif pages. These contain tabs for a Wikipedia article, seed alignments, structures, family matches, references and a curation tab. The page for the Terminator1 motif (RM00022) is shown in Figure 2 as an example. In addition to this, a tab has been added to each family page, displaying the motifs, if any, that match the family. Motif annotations for a family can be visualized by overlaying motif matches on the secondary structure image for the family. For each position of the secondary structure image, the fraction of seed sequences that match a selected motif is calculated and is represented on the structure image using a rainbow scale. Figure 3 gives an example, showing the secondary structure for the RsmY family (RF00195) overlaid with CsrA binding motif (RM00005) annotation. The interaction of RsmY with the RNA binding protein CsrA via this motif is part of a post-transcriptional regulatory network in gammaproteobacteria (23,24).

## USING RFAM 12.0 FOR GENOME ANNOTATION

With Rfam 12.0 and Infernal 1.1, users can now easily annotate RNAs in their own sequence data sets with their own computing resources. Previously, the complexity of the BLAST-based search strategy was a significant impediment to users wishing to calculate Rfam annotations on their own data, as researchers conducting a recent survey of RNAs in the pig genome reported (25). The new, simplified search strategy means that users need only to download and install Infernal 1.1 (http://infernal.janelia.org), download the Rfam 12.0 library of CMs and run Infernal's *cmsearch* program against their own sequence data sets. To exactly reproduce the method Rfam curators use when searching Rfamseq, users need to enable the --rfam, --cut_ga and --nohmmonly command line options to *cmsearch*. These options ensure that the searches run fast (--rfam), that only hits above the Rfam expertly curated score thresholds are reported (--cut_ga), and that a special HMM-only search mode that would invalidate the score thresholds is not used (--nohmmonly).

As an alternative to *cmsearch*, Infernal's *cmscan* program may be desirable to users who are interested in having search results arranged per-sequence (all Rfam families that are homologous to each sequence listed together) instead of the per-model organization (all subsequences that are homologous to each Rfam family listed together) of *cmsearch* output. However, a limitation of *cmscan* is that it will only work on sequence files for which each sequence is less than 10 Mb long (additionally, users may wish to familiarize themselves with the difference in the meaning of *E*-values between *cmscan* and *cmsearch* which is explained in the Infernal User's Guide, http://infernal.janelia.org). Currently, neither *cmsearch* nor *cmscan* carries out clan post-processing to ensure only the best scoring family match in a given clan is reported. In the future, we will provide a tool that replicates this post-processing.

To demonstrate the ability and time requirements of *cmsearch* to annotate RNAs using Rfam 12.0 in genome and metagenomic data sets, we carried out searches against 14 data sets, including a sampling of eukaryotic, bacterial and archaeal genomes (26,27), as well as metagenomics data sets of various sizes (28,29). Table 2 summarizes the search results. We searched two mammalian genomes: human and pig, which took about 650 and 450 CPU hours and annotated about 14 500 and 6200 unique regions as homologues of 796 and 625 different Rfam families, respectively. Smaller eukaryotic genomes, such as the 170 Mb *Drosophila melanogaster* genome, take about 1 day of CPU time. Bacterial and archaeal genomes, typically ranging from 2 to 5 Mb, require 30 min or less to annotate with Infernal and Rfam. Metagenomic samples require about an hour per 5 Mb of sequence. The sequencing platform used, which affects the lengths of the sequences being analysed, does not seem to have a significant impact on speed. Searches of large data sets, such as mammalian genomes or larger, can be easily parallelized, by splitting up either the Rfam library of CMs into multiple files, or the sequence data set into multiple files, or both. (Note, users who search data sets split into multiple sequence files should make sure to use the ‘-Z <x>’ command line option to *cmsearch* to define the database size as <x>, where <x> is the total size of all the sequence files being searched, in Mb. This will ensure that the statistical significance of hits (*E*-values) pertain to the full data set instead of just the file being searched.) Alternatively, users with access to a cluster can take advantage of the MPI implementation of *cmsearch* to spread the work across up to hundreds of processors. For more information on using Infernal and Rfam to annotate genomes and metagenomes, see (30,31).

**Table 2.** Summary statistics for Rfam-based annotation of RNAs in various genomes and metagenomics data sets

Genome/data set	Size (Mb)	# of hits	# of fams	CPU time (hours)	Mb/hour
*Homo sapiens*	3099.7	14 508	796	650	4.8
*Sus scrofa (pig)*	2808.5	6177	625	460	6.1
*Drosophila melanogaster*	168.7	4321	156	30	5.7
*Caenorhabditis elegans*	100.3	1022	175	20	5.2
*Saccharomyces cerevisiae*	12.2	376	96	1.7	7.3
*Escherichia coli*	4.6	256	112	0.46	10.2
*Bacillus subtilis*	4.1	211	52	0.57	7.2
*Methanocaldococcus jannaschii*	1.7	257	18	0.31	5.6
*Aquifex aeolicus*	1.6	52	7	0.22	7.3
*Borrelia burgdorferi*	0.9	44	7	0.22	4.1
Human immunodeficiency virus (HIV)	0.01	12	10	0.016	0.63
Human gut microbiome sample (sample ERS167139, 454 sequencing)	166.1	4342	54	22	7.7
Human gut microbiome sample (sample ERS235581, Illumina HiSeq sequencing) (28)	52.9	3159	47	8.5	6.2
Ocean metagenome (sample SRS580499, Illumina genome analyzer)	44.3	6692	59	13	3.5

The *cmsearch* program of Infernal 1.1 was used with Rfam 12.0 CM files and the following command-line options: --noali --cut ga --rfam --nohmmonly --cpu 0. Overlapping hits were removed such that no nucleotide was matched by more than one family by keeping the hit with the lower *E*-value in the case of overlaps (and higher bit score in the case of tying *E*-values). All searches were run as single execution threads on 3.0 GHz Intel Xeon processors. The *Homo sapiens*, *Sus scrofa*, *Drosophila melanogaster* and *Saccharomyces cerevisiae* genomes searched were obtained from Ensembl release 76 (http://www.ensembl.org/) (26) and the *Escherishia coli* (K12 substr MG1655), *Bacillus subtilis* (BSn5), *Methanocaldococcus jannaschii* (DSM 2661), *Aquifex aeolicus* (VF5) and *Borrelia burgdorferi* (CA-11 2A) genomes were obtained from release 23 of Ensembl Genomes (http://ensemblgenomes.org/) (27) for all of those the actual sequence file searched was downloaded via FTP and suffixed with .dna.toplevel.fa.gz. The HIV genome used is ENA accession AJ291720 and the four metagenomic samples were downloaded from the EBI Metagenomics Portal (https://www.ebi.ac.uk/metagenomics/) (29), and can be accessed by the sample accession listed in the table. ‘CPU time’ and ‘Mb/hour’ columns are rounded to two significant digits.

Users should bear in mind two critical caveats when attempting to annotate ncRNA genes, particularly in large eukaryotic genomes. Infernal and Rfam were designed to detect homology, but they do not attempt to discriminate between genes and pseudo-genes. In fact, these tools were designed to detect distant homology and are likely to detect much of the pseudo-gene population. Some ncRNA genes, particularly those transcribed by RNA polymerase III, are prone to generating pseudo-genes. For example, tRNAs, U6, Y RNA and 7SK all have hundreds of pseudo-genes in the human genome (32). Discriminating between genes and pseudo-genes is much harder for ncRNAs than it is for coding genes.

The second potential pitfall is that three classes of ncRNAs with internal promoters (tRNAs, 5S rRNA and SRP RNA) have formed into transposable elements called SINEs (Short Interspersed Elements) (33), numerous times over the course of eukaryotic evolution. Note that the ncRNA portion of such SINEs may still be detected by our pipeline. This is a general problem. To counter one prominent problem found in primates, the massive expansion of Alu (an SRP RNA-based SINE), the GA threshold for several SRP RNA families have been set particularly high. Thus, rather than detecting the million or so Alus in human with the SRP RNA family (RF00017), our pipeline detects only 5587 hits in human. There are only a handful of SRP RNA genes in human (34), so the remaining hits are likely to be pseudo-genes. However, artificially high GA thresholds are not a universal solution to the problem.

Another important issue related to eukaryotic ncRNA annotation is that one class of transposable elements called MITEs (miniature inverted-repeat transposable element) may confound microRNA (miRNA) gene annotation. Though MITEs are DNA transposons, they are short and imperfect inverted repeats (like miRNAs) that can be an abundant source of small RNAs. In one study, nearly one quarter of all small RNAs detected in rice derive from MITEs (35). This may lead to their curation as miRNAs, even in the absence of evidence of their being genic. However, some elements may instead represent newly evolved miRNA genes (36). As this issue is investigated further, we plan to remove any MITE-derived Rfam miRNA families which the community becomes convinced are not functional miRNAs.

## CONCLUSIONS

Rfam 12.0 includes several key steps forward for the database. The development of the new Rfam pipeline means that Rfam is now better prepared to deal with the rapid pace of sequence data generation. The family-specific nature of the pipeline allows us to run it as needed as we curate each family between releases, greatly reducing the compute required immediately prior to a release and making the release process less burdensome. Our incorporation of Infernal 1.1 means that Rfam users can now more easily calculate Rfam annotations on their own data. The data displays in Rfam have also been improved with the addition of RMfam motifs to the seed alignments and secondary structure graphics.

The new RNAcentral resource [Crossref within NAR DB issue] of ncRNA sequence data is poised to benefit Rfam, and vice versa. As of release 12.0, we provide annotations for Rfam families of the type Gene to RNAcentral and cross-links from RNAcentral to Rfam are currently available, enabling users to easily access data from both resources. The set of RNA sequences deposited in RNAcentral may ultimately be used as an additional sequence database for Rfam annotations, or possibly even as a replacement for the underlying Rfam sequence database (Rfamseq). RNAcentral data may also allow us to provide additional annotation on our families. For example, many Rfam families do not cover full ncRNA genes, but rather only a conserved region (e.g. HOTAIR conserved region 1, RF01904). The more gene-centric data in RNAcentral could allow us to highlight such families by quantifying the average fraction of a complete gene that each family covers. Finally, we expect that RNAcentral will become a common source of new Rfam entries, as novel functional RNAs are deposited there from a variety of sources. Once created, the new Rfam families will enable the discovery of homologues in other sequence collections.

Rfam continues to rely on contributions made by the community in various ways including through Wikipedia, annotations provided through our helpdesk as well as new entry submissions. We would like to thank our user community for providing such contributions, and strongly encourage others to become involved.
